# Outcomes of distal ulna locking plate in management of unstable distal ulna fractures: a prospective case series

**DOI:** 10.1007/s00402-022-04549-4

**Published:** 2022-07-18

**Authors:** K. Stock, S. Benedikt, T. Kastenberger, P. Kaiser, R. Arora, P. Zelger, J. D. Pallua, G. Schmidle

**Affiliations:** 1 University Hospital for Orthopedics and Traumatology, Medical University of Innsbruck, Anichstraße 35, 6020 Innsbruck, Austria; 2 Department for Hearing, Speech, and Voice Disorders, Medical University of Innsbruck, 6020 Innsbruck, Austria

**Keywords:** Distal ulna fractures, Distal ulna locking plate, Open reduction, Orthopedic procedures, Osteosynthesis, Case series

## Abstract

**Introduction:**

Given the absence of a satisfying plate system to deal with multifragmentary or subcapital distal ulnar fractures, the Distal Ulna Locking Plate (DUL, I.T.S. GmbH, Graz, Austria) could become a useful treatment option. This study aimed to evaluate the results of this anatomically pre-contoured plate regarding patients with unstable or displaced distal ulnar fractures.

**Methods:**

In a prospective clinical trial, 20 patients (18 female, two male; mean age 70 years (24–91 years)) with unstable or displaced distal ulna fractures between December 2010 and August 2015 were analyzed. All patients were treated with open reduction and internal fixation using the DUL. They were evaluated at three follow-up appointments at 3, 6 and 12 months postoperatively regarding their bone healing, ulnar variance (UV), range of motion (ROM) and grip strength. Patient related outcomes were measured using the Disability of the Arm, Shoulder and Hand (DASH), the Patient Rated Wrist Evaluation (PRWE) questionnaires, and the Visual Analogue Scale (VAS). The results after one year were compared to the outcome of the healthy contralateral side.

**Results:**

All fractures treated with open reduction and internal fixation using the Distal Ulna Locking Plate healed within 6 months and showed stable ulnar variances after surgery. ROM (rotational plane 81.1 ± 9.0°, sagittal plane 55.1 ± 14.6°, frontal plane 33.0 ± 9.4°) and grip strength (18.7 ± 7.1 N) at the follow-up after 12 month had similar values compared with the uninjured side. The mean DASH score (36.4 ± 29.0), the PRWE-score (14.5 ± 27.0), and the VAS (at rest 0.5 ± 1.1, during activity 1.2 ± 2.4) after one year had no significant difference to the uninjured side. The surgeon’s overall satisfaction rate regarding plate handling reached 81.8%.

**Conclusion:**

Stabilization of unstable distal ulna fractures using the DUL restores nearly normal anatomy and function. Its pre-countered design, volar placement, and enhanced stability present a satisfying plate system.

**Trial registration:**

The trial was retrospectively Registered at www.clinicaltrials.gov on 16 December 2021 (Trial Registration Number: NCT05329012).

## Introduction

The distal ulna and the distal radioulnar joint (DRUJ) are essential structures for forearm rotation. Loss of supination and pronation impedes the function of the upper limb and, subsequently, activities of daily living. The restoration of forearm rotation depends on accurate fracture reduction and early postoperative motion exercises [1, 2]. Functional deformity follows in a significant loss of motion of the forearm, chronic pain, and instability [3].

Isolated distal ulna fractures are relatively uncommon injuries, and there are few reports on fractures of the lower end of the ulna without concomitant fractures of the radius [4]. Up to 6% unstable metaphyseal fractures are seen in patients with unstable distal radius fractures [5]. The two main options to manage these fractures are immobilization in an above-elbow cast or open reduction and internal fixation (ORIF) [1, 2].

Many distal ulna fractures are considered stable after reduction and stabilization of concomitant distal radius fractures and can achieve satisfactory outcomes with conservative treatment [6]. Especially elderly patients are mostly treated conservatively with plaster casts because of their poor bone quality and often observed failures of conventional osteosynthesis. But conservative treatment also causes well-known complications with prolonged immobilization leading to muscle atrophy and joint stiffness resulting in an inability to return to previous activity levels [7].

In displaced or unstable ulna fractures, poor outcomes after non-operative therapy have been reported [8, 9]. Limited data have been published regarding the management of ulnar head and neck fractures [3, 6, 10] and the outcomes following operative treatment of these injuries [11]. ORIF of the distal ulna fracture may provide stable fixation, early motion, and rehabilitation, but accurate reduction and retention of the fracture in the distal ulna is challenging with its variable and curved shape [4]. The typically small and often osteoporotic fracture fragments and the small non-articular arc of the ulna head limit hardware placement making internal fixation technically challenging [3, 12].

Several surgical techniques have been proposed to obtain fixation, including angular-stable implants like minicondylar blade plates [3] or minifragment locking plates [12], locked 2.0 mm (mm) plates, or percutaneous pinning [3, 13]. But still, proper anatomic reconstruction of the ulnar head may not be achieved [12]. So far, there are no satisfying plate systems for unstable distal ulna fractures to address the ulnar head. Most of the products have a dorsal plate position on the ulna leading to irritation of the extensor tendons [14].

Anatomically pre-contoured plate designs may help to address distal ulna fractures and avoid salvage procedures. The present study evaluated the application and possible complications of a newly introduced plate design treating unstable distal ulna fractures and the surgeons' experiences with its application.

## Materials and methods

**Fig. 1:**
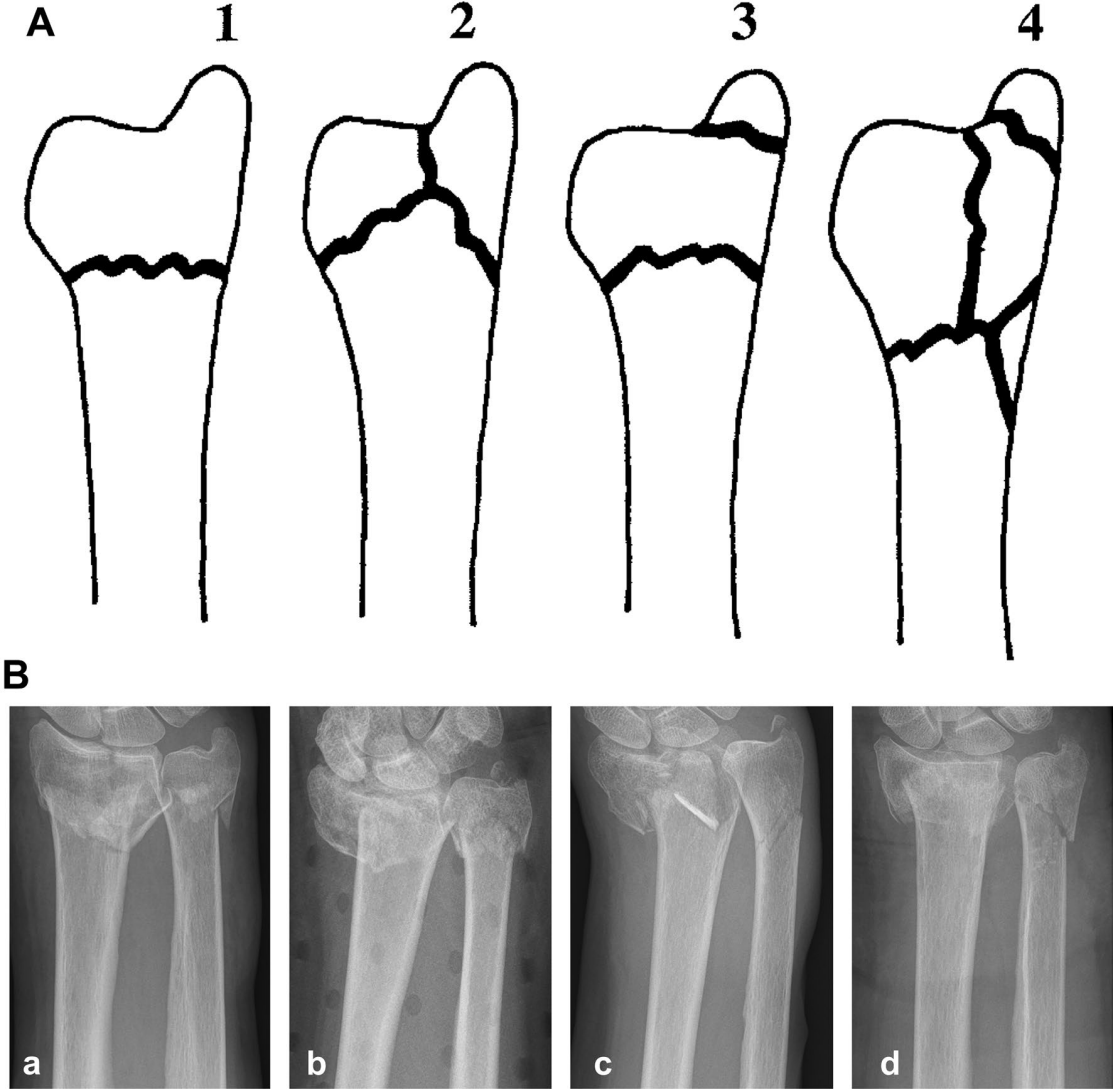
**A** Fracture classification of the distal ulna according to Biyani et al. [5]. Type 1: Extra-articular fractures with minimal comminution (10 patients). Type 2: Fractures of the metaphysis and the ulnar styloid (1patient). Type 3: Ulna fracture combined with fracture of the ulnar styloid (6 patients). Type 4: Completely comminuted fracture of the ulna metaphysis (3 patients). **B** Anterior–posterior radiographs showing distal ulna fractures according to Biyani classification 1–4 (**a**–**d**)

A prospective clinical trial of patients with unstable or displaced distal ulna fractures between December 2010 and August 2015 was conducted. Patients younger than 18 years, with stable ulna fractures, ulna shaft fractures, previous pathology on the affected wrist, and patients with drug- or alcohol addiction or high risk of anesthesia were excluded. In this series, 20 patients (18 female, two male) with a mean age of 70 years (24–91 years) with unstable or displaced distal ulna fractures were included. Both the right and left ulna were affected 10 times. 3 patients suffered from isolated ulna fractures and 17 patients from distal forearm fractures. Injuries occurred due to falls (16 times), domestic accidents [2], sports trauma (1-time) or car crash (1-time). Distal ulnar fractures were classified according to the Biyani classification [5]. It distinguishes 4 Types according to fracture patterns and anatomical location (Fig. 1). There were 3 open fractures (I°).

All patients were treated with ORIF using the Distal Ulna Locking Plate (DUL, I.T.S. GmbH, Graz, Austria) at a mean of 8 days (range 3 to 19 days) after injury.

### Implant description

The DUL has an anatomically pre-contoured design to fit the distal ulna. It aims for good soft tissue coverage by volar placement combined with enhanced stability using locking screws. The plate has two main parts, one for the ulnar head and one for the shaft. The distal part of the implant envelops the ulnar head in a half-open cup-like shape from volar to ulnar. It allows for multi-directional screw placement in the short ulnar head fragment, increasing the stability of the construct. The head component of the plate accepts multi-directional (15°) locking screws for angular stability, while the shaft component receives both locking and non-locking cortical screws for dynamic compression and improved length adjustment.

The titanium plate is available in a left and right version, both in a wide or small model and in 3 different lengths [15]. Indications for using the DUL are (multifragmentary or subcapital) fractures of the ulnar head, comminuted metaphyseal fractures of the distal ulna, or combined ulnar head and ulnar shaft fractures [15].

### Surgical technique

The procedure was performed under general anesthesia or a brachial plexus block by six different surgeons of the trauma surgery department. The patients were placed in the supine position with the injured limb on a hand table. A tourniquet was used in 13 patients. All open fractures underwent debridement and irrigation. For fixation of the ulna, the arm was positioned in full supination on a supporting roll in slight elbow flexion or vertically in neutral rotation according to the surgeon’s preference. A longitudinal incision was made along the ulnar border of the forearm starting at the ulnar styloid running 5–7 cm proximal. 17 cases with both bone forearm fractures were treated with volar plate fixation (10 patients with Variable Angle LCP volar distal radius plate 2.4 mm–Synthes, and 7 patients with Distal radius fracture plate 2.5 mm—Medartis Aptus) using a separate Henry approach. In all patients, the DRUJ was stable after internal fixation.

**Fig. 2:**
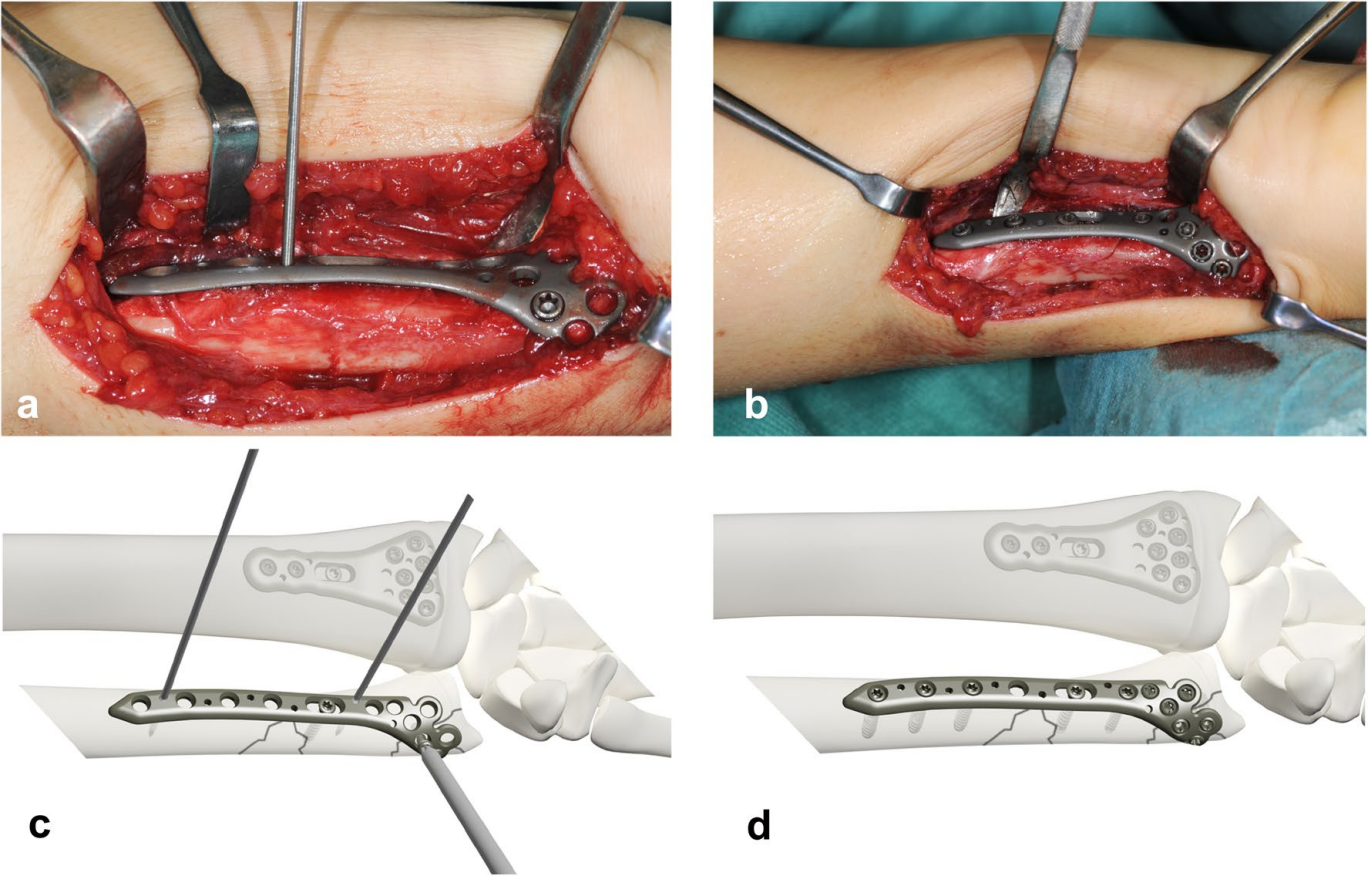
**a** Longitudinal skin incision along the ulnar border of the forearm starting at the ulnar styloid. The pronator muscle is detached from the periosteum to preserve it. The fracture is reduced, the plate is applied with provisionally securing with K-wires. **b** The plate is fixed with cortical screws in the ulnar shaft and monocortical locking screws in the ulnar head. Exemplary drawing of the distal forearm in a supinated position, fracture line indicated. Distal radius plate attached, DUL secured with K-wires (**c**) and definitive screw placement (**d**)

Special care was taken to preserve the superficial branch of the ulnar nerve that may cross the surgical field in the distal part of the approach. The pronator quadratus muscle was detached ulnarly and retracted radially, preserving the periosteum. The fracture was reduced and provisionally secured with K-wires (Fig. 2a). The plate was positioned under fluoroscopic control on the palmar surface of the ulnar shaft and temporarily fixed with K-wires. Then the first screw was put into the oblong hole, and after final length adjustments, the remaining cortical screws in the shaft were inserted. The ulnar head was fixed with at least 3 monocortical locking screws, respecting the integrity of the distal radioulnar joint. Multi-directional screw placement from palmar and ulnar contributed to overall stability (Fig. 2b).

After meticulous hemostasis wound closure was performed in two layers, a forearm splint was applied until suture removal 10–14 days after surgery. The postoperative rehabilitation protocol included short-term immobilization of the wrist in a plaster cast or bandage until suture removal with immediate forearm rotation exercises. Active wrist mobilization was initiated from 3 to 6 weeks after surgery, depending on the surgeon’s preference. Patients were instructed to limit forearm rotation for the first 4 weeks to 40 degrees of supination and pronation, respectively.

### Evaluation

The patients were evaluated at follow-up appointments postoperatively after 3, 6, and 12 months. The patients were retrospectively evaluated for time to union, amount of shortening, range of motion (ROM), grip strength, pain, and graded with the Disability of Arm, Shoulder, and Hand (DASH) scoring system and the Patient Related Wrist Evaluation questionnaire (PRWE). The results after one year were compared to the outcome of the healthy contralateral side.

In standardized posteroanterior radiographic projection of the wrist, the ulnar variance (UV) of the distal radius was measured using the perpendicular line method [16]. Furthermore, lateral radiographs were used additionally to evaluate bone healing. A handheld goniometer was used to assess the forearm and wrist’s ROM at every follow-up (Biometrics Ltd., SN: M132 2011-09) [17]. Forearm pronation and supination, wrist flexion, extension, and ulnar and radial deviation were measured with the elbow in 90° of flexion and the arm at the patient’s side. As typical values, angles of 70° for flexion and extension, 20° for radial deviation, 40° for ulnar deviation were applied [18]. The patient’s ability to close the injured fist thoroughly was verified. Grip strength was measured using a dynamometer (Biometrics Ltd., SN: M13069 2011-06). The contralateral, unaffected forearm and wrist were used as controls for each measurement.

A visual analogue scale (VAS) assessed pain at rest and during activity. This scale ranges from 0 to 10, with 0 points representing “no pain” and 10 points “worst possible (imaginable) pain” [19]. All values were compared to the unaffected side. Two validated, patient-based subjective questionnaires were used to assess patient-reported functional outcomes: the DASH quantifies disabilities related to the upper extremity, with lower scores representing less pain and disability. A score of 0 points indicates a perfectly functioning upper extremity, whereas a score of 100 indicates complete impairment [20, 21]. PRWE [22] is a joint-specific questionnaire that enquires about symptoms of the wrist (presence, intensity, and frequency of pain) and functional limitations concerning activities of daily living (ADL). The response scale is numeric from 0 to 100 points (0 = best, 100 = worst rating) [23].

A standardized feedback form was used to gather information about the intraoperative performance of the plate system from the surgeons. The surgeon’s satisfaction was conducted using a 3-point scale: satisfying (score 1), less satisfying (score 2), and not satisfying (score 3). Furthermore, complications with implant positioning, implant fixation, and handling were documented, and a 3-point scale evaluated the management of plate and screw positioning and the overall handling: very easy (score 1), easy (score 2), and difficult (score 3).

### Statistical analysis

Statistical analysis was performed using statistical software (IBM Deutschland GmbH, SPSS Statistics 25). The variables were tested for normality using the Kolmogorov–Smirnov test and have been reported as mean ± standard deviation (SD). The variables were compared using Student’s *t*-test for parametric data. Significance was set at a *p*-value less than 0.5.

## Results

All fractures healed. Hardware removal was performed in 3 patients because of discomfort. There were no infections, no hardware failures, screw loosening or loss of reduction. The average follow-up time was 14 months (range 12–27 months).

### Radiographs

UV after injury (2.5 ± 2.3 SD) and 12 months after the accident (1.0 ± 2.5 SD) showed no significant difference compared to the uninjured wrist (*p* = 0.882). The average UV was, 1.3 ± 2 SD after reduction, 0.2 ± 2.1 SD after 3 months, and 0.4 ± 2.7 SD after 6 months. After 3 months, 90% of 19 fractures were healed radiographically. All fractures healed within 6 months.

### Objective outcomes

The mean degrees ± SD of all ROM measurements and grip strength are presented in Table 1.

**Table 1:** Functional outcome parameters (ROM, grip strength) were obtained in the injured arm at the respective follow-ups and the uninjured side

	Uninjured side	3 months	6 months	12 months
ROM mean ± SD				
Forearm pronation	87.1 ± 4.8	69.169 ± 15.8^a^	74.0 ± 7.9^a^	80.8 ± 9.2^a^
Forearm supination	86.7 ± 6.1	70.8 ± 14.9^a^	76.0 ± 16.3^a^	81.8 ± 8.8^a^
Wrist extension	65.3 ± 15.7	45.3 ± 16.2^a^	51.9 ± 16.7^a^	53.3 ± 16.9^a^
Wrist flexion	55.9 ± 12.8	39.6 ± 8.7^a^	44.3 ± 10.1^a^	50.5 ± 11.6
Wrist ulnar deviation	35.8 ± 9.3	31.9 ± 10,5^a^	31.7 ± 9.4^a^	36.9 ± 9.0
Wrist radial deviation	22.0 ± 8.1	18.7 ± 6,5^a^	23.3 ± 7.7^a^	27.8 ± 8.3
Grip strength mean N ± SD	23.275 ± 7.6	13.7 ± 6.8^a^	16.7 ± 7.9^a^	18.7 ± 7.1

The values of 20 analyzed patients are given as mean and standard deviation

*ROM* range of motion; *SD* standard deviation, *N* Newton

^a^Indicating a statistically significant difference (*p* < 0.05) in comparison to the values on the healthy side

The injured side showed lower values than the uninjured side in all measurements at all follow-up appointments. From 3 to 12 months the range of forearm pronation (*p* = 0.001) and supination (*p* = 0.003), wrist flexion (*p* = 0.000) and radial deviation (*p* = 0.000) increased significantly, while wrist extension (*p* = 0.131), ulnar deviation (*p* = 0.093) and grip strength (*p* = 0.260) did not show statistically significant change. After 3 months, 17 of the participating patients were able to close their injured fist completely. This number increased 6 months after surgery to 18 and in the 1-year-control to 19. In each degree of freedom ROM approximated to values to the healthy, uninjured side presented in Table 1.

There was no significant difference in grip strength after 12 months (*p* = 0.3) compared to the healthy side, in contrast to the 3 month (*p* = 0.039) and 6 month (*p* = 0.037) follow-up with significantly less strength.

### Subjective outcomes

Table 2 presents the results in outcome parameters evaluated from DASH and PRWE questionnaires and VAS. Similar outcomes showed the evaluation of PRWE-scores: A negative correlation was seen at the 3 months (23.8 ± 20.5) and 6 months (22.5 ± 28.4) control, while Scores after one year were not significantly different from those of the uninjured side. The DASH-scores declined continuous to 12 months follow-up.

**Table 2:** Subjective outcome parameters (questionnaires and pain level) were obtained in the injured arm at the respective follow-ups and the uninjured side

	Uninjured side	3 months	6 months	12 months
DASH mean ± SD	1.5 ± 5.03	23.16 ± 22.18^a^	17.1 ± 21.5^a^	15.9 ± 27.3^a^
PRWE mean ± SD	3.6 ± 15.7	23.8 ± 20.5^a^	22.5 ± 28.4^a^	14.5 ± 27.0
VAS mean ± SD				
At rest	0.0 ± 0.0	0.9 ± 1.5^a^	0.8 ± 1.7^a^	0.5 ± 1.1
During activity	0.18 ± 0.59	2.5 ± 2.7^a^	2.6 ± 2.8^a^	1.2 ± 2.4

The values of 20 analyzed patients are given as mean and standard deviation

*DASH* Disability of the Arm, Shoulder, and Hand; *SD* standard deviation; *PRWE* Patient Rated Wrist Evaluation; *VAS* Visual Analogue Scale

^a^Indicating a statistically significant difference (*p* < 0.05) in comparison to the values on the healthy side

Pain-scores decreased progressively (Table 2).

### Surgeon’s satisfaction

The overall satisfaction rate reached 81.8%. According to the categorical ratings, a satisfying plate handling was reported in 18 patients while there were some difficulties with plate positioning in 9 patients. Apart from issues with plate fixation in 2 patients, no complications occurred. The handling of plate and screws was reported to be easy or very easy.

## Discussion

Fractures of the distal ulna are often associated with fractures of the distal radius with many injuries [24]. Most fractures can be treated conservatively, but malaligned or unstable fractures may compromise radioulnar function and need to be treated surgically [3].

Anatomic fixation of distal ulna fractures remains technically challenging [6]. Therefore, several surgical methods have been described, including percutaneous K-wires [5], Herbert-screw insertion [25], condylar blade plating [3], ulna hook plate fixation [6, 26], and locked plating [12]. Each procedure has benefits and disadvantages, depending on the fracture pattern and external conditions, and is selected according to fracture type and surgeons’ experience.

Standard plates will only achieve fixation with one or two screws in the distal fragment. The screws cannot be bicortical because of the proximity of the joint [3]. This makes unique plate designs necessary to achieve favorable and reproducible results. Locked fixation can achieve stable fixation in unstable, osteoporotic, and comminuted fractures. Therefore, late complications can be minimized, and the function of the wrist and forearm can be preserved [12]. As Lee et al. suggest, an indirect reduction method using stable angular implants can be used to perform stable fixation of relatively small fragments [6].

The locked plate fixation first introduced by Dennison et al. for the treatment of distal ulna fractures achieved good outcomes [12]. However, it has some limitations. Fixation and restoration of these plates is difficult if the fracture extends to the intraarticular neck or head portion, because the articular surface of the fixation area is not large enough.

In the investigations of Ring et al. [3] using a condylar plate fixation for ulna fractures in 24 patients, alignment and healing were achieved in most cases with good functional results. The plate they used was primarily intended to fix periarticular fractures of the metacarpals and proximal phalanges to secure unstable fractures of the distal ulna. It allows stabilizing the fracture fragments via stable angular fixation and a blade. Another benefit of the plate is that it can be rotated to fit nicely on the bone. For this reason, the plate needs to be contoured to fit along the diaphysis of the ulna [3].

DUL is designed to meet the mechanical needs and fit the distal ulna’s shape. Contouring the plate is possible due to the multi-directional and locking screw options but is rarely necessary.

In the study of Gschwentner et al., different surgical treatment options were used to address unstable distal forearm fractures. Participants treated with ORIF on radius and ulna showed decreased ROM, due to slight malrotation with rigid fixation or impingement of the ulnar plate at the DRUJ at rotation [27]. In our study using the DUL these problems of plate positioning did not occur.

Our study shows that satisfying outcomes can be achieved in distal ulna fractures with the DUL. It is designed to achieve good soft tissue coverage in the shaft region by volar placement and to engulf the ulnar part of the ulna head with its cup like, anatomically pre-contoured design. Rigid stability is achieved also in osteoporotic bone using multi-directional locking screws in the ulna head area enabling stable plate positioning. The subcutaneous location of the ulna requires a low-profile implant to minimize symptoms from major hardware [12]. The volar positioning of the DUL beneath the muscle decreases the requirement of hardware removal. Its distal cup-like shape envelops the volar and ulnar aspect of the ulnar head increasing stability by buttressing of fragments and adding fixation options.

All patients achieved good function in their arms and wrists, with only slight restrictions noted in forearm pronation and supination. These results in the ROM are comparable to those of Ring et al. and Dennison et al. [3, 12]. A nearly equal extent in ROM compared to the healthy side suggests that fixation with DUL does not strongly restrict the patient’s mobility. Because of rigid fracture fixation, the early motion of the forearm can be allowed. This is, in consequence, helpful for the recovery of forearm function.

In distal forearm fractures both, the osteosynthesis of the distal radius as well as the distal ulna, impact on the restoration of stability of the DRUJ [28, 29]. While the stabilization of the distal radius is well documented to achieve good results with modern plating systems, the osteosynthesis of the distal ulna can be challenging in distal forearm fractures. As the distal radius was corrected adequately in all cases, we believe that this concomitant injury did not distort our results on the plating of unstable distal ulna fractures.

The injured arm had no significantly reduced grip strength after 12 months (*p* = 0.3) compared to the healthy side. Significant progress in grip strength, with the trend to converge regular values were achieved. This is in accordance with several reports that show better functional results after 3 and 6 months in distal radius fractures [30, 31].

Pain-scores decreased progressively from week 12 (mean 0.9 at rest and 2.5 during activity) to postoperatively after one year (mean 0.5 at rest and 1.2 during activity range).

The mean DASH score of our study group (36.5 ± 29.0 points) was well within the range of the average population [32]. No patients noted restrictions in their work, sports, or daily activities one year after injury. Equal results are reached in evaluation of the PRWE-scores in our population. The mean PRWE-score was 14.5 of possible 100 points after one year.

The overall satisfaction of plate handling reached 81.8%, inappropriate handling did not occur. As Henle et al. described, several circumstances, such as osteoporotic bone, comminuted fractures, or a specific fracture localization, can affect plate positioning and handling [32].

Possible disadvantages of the DUL are surgical exposure associated with a subsequent scar.

Complications like neuropraxia of the dorsal sensory branch of the ulnar nerve, described by Dennison et al., did not occur in our study population [12]. Beneficially, no cast immobilization is required compared to intramedullary K-wires, showing better functional results.

This study had some limitations. The primary limitation is the small number of patients, with the result that comparative groups were missing. Multiple surgeons treated the patients involved. Furthermore, heterogeneous groups of fractures were analyzed, so the comparability of results was impaired. Third, the majority of participants was female, however, this matches with regular gender distribution in distal ulna fractures.

## Conclusion

The current study results showed that restoration of ulna length, rotational alignment, and interosseous space between the radius and the ulna could reliably be achieved with the anatomically pre-contoured DUL. Because of the good clinical and radiological results, we concluded that its pre-countered design, volar placement, and enhanced stability present a satisfying plate system. This procedure exhibited satisfactory clinical outcomes and uncomplicated implant handling.

## Abbreviations

ADL: Activities of daily living
DASH: Disability of arm, shoulder, and hand scoring system
DRUJ: Distal radioulnar joint
DUL: Distal Ulna Locking Plate
K-wires: Kirschner-wires
Mm: Millimeter
mmHg: Millimeter of mercury
N: Newton
ORIF: Open reduction and internal fixation
PRWE: Patient related wrist evaluation questionnaire
ROM: Range of motion
SD: Standard deviation
UV: Ulnar variance
VAS: Visual analogue scale
